# Elastin haploinsufficiency accelerates age-related structural and functional changes in the renal microvasculature and impairment of renal hemodynamics in female mice

**DOI:** 10.3389/fphys.2023.1141094

**Published:** 2023-04-25

**Authors:** Alethia J. Dixon, Patrick Osei-Owusu

**Affiliations:** Physiology and Biophysics, School of Medicine, Case Western Reserve University, Cleveland, OH, United States

**Keywords:** renal microvasculature, elastin, aging, renal hemodynamics, renal autoregulation

## Abstract

Age-related decline in functional elastin is associated with increased arterial stiffness, a known risk factor for developing cardiovascular disease. While the contribution of elastin insufficiency to the stiffening of conduit arteries is well described, little is known about the impact on the structure and function of the resistance vasculature, which contributes to total peripheral resistance and the regulation of organ perfusion. In this study, we determined how elastin insufficiency impinges on age-related changes in the structure and biomechanical properties of the renal microvasculature, altering renal hemodynamics and the response of the renal vascular bed to changes in renal perfusion pressure (RPP) in female mice. Using Doppler ultrasonography, we found that resistive index and pulsatility index were elevated in young *Eln* ^+/−^ and aged mice. Histological examination showed thinner internal and external elastic laminae, accompanied by increased elastin fragmentation in the medial layer without any calcium deposits in the small intrarenal arteries of kidneys from young *Eln* ^+/−^ and aged mice. Pressure myography of interlobar arteries showed that vessels from young *Eln* ^+/−^ and aged mice had a slight decrease in distensibility during pressure loading but a substantial decline in vascular recoil efficiency upon pressure unloading. To examine whether structural changes in the renal microvasculature influenced renal hemodynamics, we clamped neurohumoral input and increased renal perfusion pressure by simultaneously occluding the superior mesenteric and celiac arteries. Increased renal perfusion pressure caused robust changes in blood pressure in all groups; however, changes in renal vascular resistance and renal blood flow (RBF) were blunted in young *Eln* ^+/−^ and aged mice, accompanied by decreased autoregulatory index, indicating greater impairment of renal autoregulation. Finally, increased pulse pressure in aged *Eln* ^*+/−*^ mice positively correlated with high renal blood flow. Together, our data show that the loss of elastin negatively affects the structural and functional integrity of the renal microvasculature, ultimately worsening age-related decline in kidney function.

## Introduction

Age-related remodeling of the arterial vasculature often manifests as decreased compliance and increased stiffness and is most notable in conduit vessels, including the aorta and carotid arteries (Greenwald, 2007; Avramovski et al., 2014; Vallee et al., 2020; Zhang et al., 2020). Vascular stiffening is attributable to altered collagen-to-elastin ratio in the extracellular matrix (ECM), including the elastic lamina (Arribas et al., 2006; Wagenseil and Mecham, 2012; Halper, 2018). The production of these two ECM proteins is developmentally regulated to ensure appropriate vascular compliance in conduit arteries necessary for biomechanical buffering of the high pulsatile flow of ejected blood from the left ventricle into the circulation (Wagenseil and Mecham, 2009). Because elastin gene expression ceases shortly after postnatal development but is continuously degraded, albeit slowly, collagen-to-elastin ratio in the vessel wall continues to increase with age. This leads to decreased vascular compliance that manifests as elevated pulse pressure and increased pulse wave velocity (Faury et al., 2003; Wagenseil and Mecham, 2009; Le et al., 2011). Elastin degradation with aging leads to the gradual loss of functional elastin and is hallmarked by the thinning and disorganization of vascular elastic laminae, particularly elastic fibers in the medial layer (Li et al., 1998a; Li et al., 1998b). As such, a cross section of an aged artery may appear to have paradoxically more lamellar units relative to a younger artery (Faury et al., 2003). These age-related structural changes in the vascular ECM are not limited to elastic arteries but are also evident in the small arteries and arterioles of the microcirculation, including those in the mesenteric and renal vascular beds (Osei-Owusu et al., 2014; Owens et al., 2017). Literature on the structural and functional consequences of age-related stiffening of conduit vessels is extensive (Izzo and Mitchell, 2007; Pezet et al., 2008; Chung et al., 2009). In contrast, relatively less is known about the functional relevance of elastin in microvessels and the impact of age-related remodeling of the microcirculation on visceral organ perfusion, particularly the kidney, which plays a key role in the long-term regulation of blood pressure by maintaining water and electrolyte homeostasis.

Previously, we used a murine model of elastin haploinsufficiency (*Eln* ^*+/−*^ mice) to demonstrate that elastin insufficiency alters the structure and function of the renal microvasculature and that these changes precede the development of hypertension (Owens et al., 2017). In the same study, we also found that elastin insufficiency is associated with augmented renal vascular resistance and low renal blood flow in adult animals (Owens et al., 2017). Overall, this work indicated that elastin insufficiency by itself can affect renal structure and function, leading to kidney dysfunction. As aging is also associated with high prevalence of chronic kidney disease, we sought to determine whether elastin haploinsufficiency predisposes *Eln* ^*+/−*^ mice to accelerated decline in kidney function with age.

Thus, our goal in this study was to examine how elastin insufficiency impinges on age-related changes in the structural and biomechanical properties of the renal microvasculature, thereby affecting renal hemodynamics and the response of the renal vascular bed to changes in renal perfusion pressure in 4–6-month and 14–16-month-old *Eln* ^*+/+*^ and *Eln* ^+/−^ female mice. Our findings give a novel insight into the impact of stiffened renal vascular bed *per se* on the autoregulation of renal blood flow, thus further enhancing our understanding of age-related decline in kidney function.

## Materials and methods

### Animals

Studies were performed in accordance with protocols approved by the Institutional Animal Care and Use Committee (IACUC) of Case Western Reserve University, School of Medicine. Experiments were performed using young (4–6-month-old) and aged (14–16-month-old) female wild-type (*Eln* ^+/+^) and elastin heterozygous (*Eln* ^+/−^) mice that have been backcrossed more than ten generations into the C57BL/6 genetic background (Charles River). The generation of *Eln* ^+/−^ mice has been described previously (Li et al., 1998a; Faury et al., 2003). Mice were provided access to food and water *ad libitum* in our institution’s animal resource center at 22°C with a 12-h light/dark cycle.

### Pulse wave Doppler ultrasonography of renal artery

Renal blood flow waveforms were acquired in isoflurane-anesthetized adult female mice using high-frequency ultrasound Vevo 3100 (FUJIFILM VisualSonics, Ontario, Canada). Images of renal arteries were acquired in B-mode with color Doppler setting to identify arterial blood flow. Peak systolic velocity (PSV), least diastolic velocity (LDV), and mean velocity (MV) determined from the renal Doppler waveforms were used to calculate resistive index ( $RI=\frac{\left(PSV-LDV\right)}{PSV}$ ) and pulsatility index ( $PI=\frac{\left(PSV-LDV\right)}{MV}$ ), as previously described (Warshauer et al., 1988; Salgado et al., 1999; Degirmenci et al., 2013; Jie et al., 2016; Koch et al., 2019). Each parameter was calculated in triplicate using three separate waveforms.

### Measurement of renal blood flow

Renal blood flow (RBF) experiments were performed as previously described (Osei-Owusu et al., 2015). In brief, mice were anesthetized with 1.5% isoflurane, and a catheter filled with 10% heparin-saline solution was placed in the right carotid artery. A PE-10 catheter was placed in the left external jugular vein for fluid and drug delivery, followed by the placement of a PE-90 bladder catheter for urine collection via ventral abdominal incision. One piece each of 6–0 silk ligature was placed loosely around the celiac and the superior mesenteric arteries. For RBF measurement, a 0.5PSB perivascular nanoprobe (Transonic, Ithaca, NY) was placed on the left renal artery. Shortly after the surgical procedures were completed, baseline blood pressure (BP) and RBF were recorded for a few minutes prior to a bolus intravenous injection of phosphate-buffered saline cocktail (PBS; 8 μL/g body weight) containing 2% bovine serum albumin, 1 μg/mL norepinephrine, 0.5 ng/mL arginine vasopressin, 0.2 mg/mL hydrocortisone, and 0.2 μg/mL aldosterone, followed by continuous infusion of the same solution at 0.5 μL/min/g body weight. Infusion of the cocktail was steadily maintained for 30 min to allow for equilibration. Then renal perfusion pressure was increased by simultaneously tightening the ligatures around the celiac and mesenteric arteries, while BP and RBF were continuously recorded for 20 min. Hemodynamics data were recorded and analyzed with LabChart 8 (ADInstruments, Colorado Springs, CO). The bladder was continuously emptied via an implanted catheter, as previously described (Osei-Owusu et al., 2015; Owens et al., 2017).

### Histological analysis

Kidneys harvested from terminal renal hemodynamics experiments were cut into two in cross section and fixed in 10% formalin solution. Tissue embedding was performed at the Tissue Procurement Core at University Hospitals, Cleveland, OH, and sectioning and histological staining were performed at Cleveland Clinic, Cleveland, OH. Kidneys were stained with Movat pentachrome dye for elastic fibers and Von Kossa dye for the presence of calcium deposits. Scoring for elastin fiber fragmentation was achieved by blinding participants who scored the vessels based on the fragmentation on a scale of 1-3, with 3 being the highest abundance of fragmentation. Vascular calcification was then evaluated histomorphometrically at ×100 magnification in bright field. Double-blind assessment of vascular calcification was performed on random sections of aorta by separate investigators. The degree of Von Kossa positivity was scored semi-quantitatively, with scores ranging from 0 to 3, depending on the surface of Von Kossa positivity. A score of 0 indicated no Von Kossa positivity; a score of 1, focal Von Kossa positivity, larger than or not overlying a cell nucleus; a score of 2, partially circumferential Von Kossa positivity in the tunica media of the vessel; and a of score of 3, Von Kossa positivity in the tunica media spanning the complete circumference of the vessel.

Histological measurements were completed using NIH ImageJ software. Measurements were obtained by manually tracing the internal perimeter (lumen) and external perimeter of the vessels. The internal (D_i_) and external (D_e_) diameters were calculated using the following equation: D=Cπ , where C is the traced perimeter of the vessels. Wall thickness (WT) was determined with the following equation: $WT=\frac{\left(D_{e}-D_{i}\right)}{2}$ . To estimate the thickness of the vessel wall relative to the lumen, wall thickness/lumen ratio was calculated as a percentage.

### Renal interlobar artery isolation

Kidneys harvested from young and aged *Eln* ^+/+^ and *Eln* ^+/−^ mice following RBF measurements were split into two sagittal halves. One-half of the kidney was secured with pins for vessel isolation in a cooling chamber containing chilled Ca^2+^-free physiological saline solution (PSS) as previously described (Owens et al., 2017). The composition of the PSS is as follows (in mM): 140 NaCl, 5 KCl, 1.2 MgSO_4_, 10 NaAcetate, 10 HEPES, 1.2 Na_2_H_2_PO_4_, 5 glucose, 3 mM ethylene glycol-bis (β-aminoethyl ether)-N,N,N′,N′-tetraacetic acid (EGTA) with a pH of 7.4. The renal medulla was removed to visualize the renal microvascular tree, including segmental, interlobar, and arcuate arteries. After careful dissection and excision, interlobar artery segments were mounted on glass pipettes in a pressure myography chamber (Living Systems, St Albans City, VT) and secured with two pieces of silk ligature at each end of the vessel. Both the vessel lumen and the vessel chamber were filled with Ca^2+^-free PSS, while the bath was warmed to 37°C before the start of the experiment. Intraluminal pressure and vessel bath temperature were maintained by servo-controlled pressure pump and temperature control systems, respectively. Vessels that failed to hold consistent pressure were considered leaky and were discarded. To measure passive loading diameter, intraluminal pressure was increased from 40 to 160 mmHg in 20-mmHg increments and maintained at each pressure for 1 min. To measure passive unloading diameter, intraluminal pressure was decreased from 160 to 40 mmHg in 20-mmHg decrements and maintained at each pressure for 1 min.

### Examination of biomechanical properties of renal interlobar arteries

Incremental distensibility (ID; %/mmHg), which represents the percent change in arterial internal diameter for each mmHg change in intraluminal pressure, was calculated as follows: $\left[\frac{\left(D_{1}-D_{0}\right)}{\left(D_{1}*\Delta P\right)}\;100\right]$ , where D_0_ represents the internal diameter before increasing pressure, D_1_ is the internal diameter after increasing pressure, and ΔP is the change in intraluminal pressure which is 20 mmHg, as previously described (Jie et al., 2016).

### Statistical analysis

Statistical analysis was performed using Prism 9 (GraphPad Software; San Diego, CA). Multiple comparisons were performed using analysis of variance (ANOVA) followed by Sîdak *post hoc* test where appropriate. All values are presented as mean ± standard error of the mean (s.e.m). A *p*-value <0.05 was considered statistically significant.

## Results

### Elastin haploinsufficiency increases impedance to renal blood flow

**FIGURE 1:**
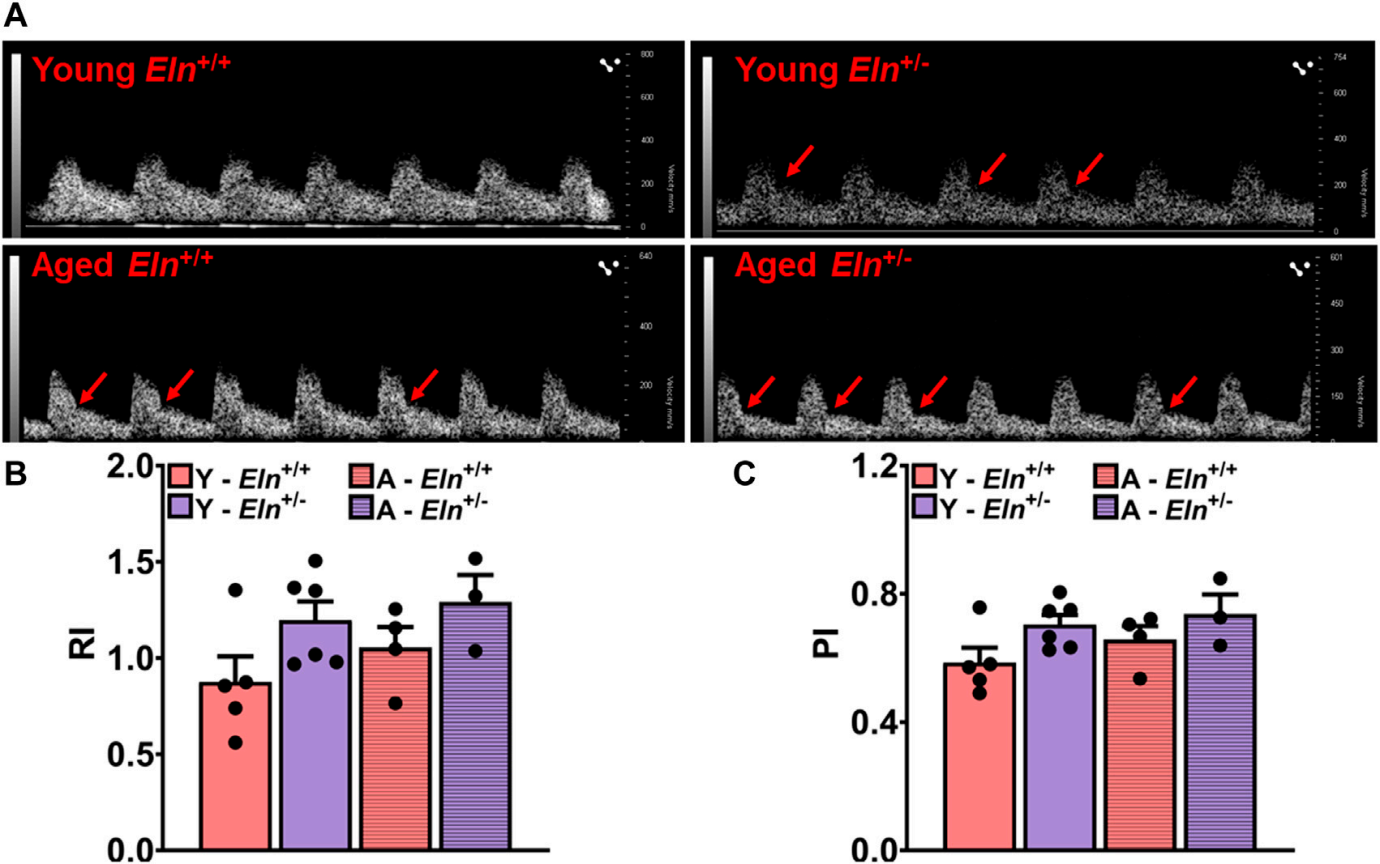
Doppler ultrasonography of renal arteries in anesthetized young and aged *Eln* ^+/+^ and *Eln* ^+/−^ mice. **(A)** Representative waveforms of renal artery Doppler ultrasound of young and aged *Eln* ^+/+^ and *Eln* ^+/−^ mice. Systolic notches (red arrows) can be seen in the waveforms of *Eln* ^+/−^ mice of both age groups as well as in aged *Eln* ^+/+^ mice. **(B)** Average resistive index, $RI=\left(\frac{PSV-EDV}{PSV}\right)$ of *Eln* ^+/+^ and *Eln* ^+/−^ mice of both age groups (*n* = 3–6 per group) was derived from peak systolic (PSV) and end diastolic (EDV) velocities of renal Doppler waveforms represented in 1A. **(C)** Average pulsatility index, $PI=\left(\frac{PSV-EDV}{MV}\right)$ of *Eln* ^+/+^ and *Eln* ^+/−^ mice of both age groups was derived from peak systolic, end diastolic, and mean (MV) velocities of renal Doppler waveforms represented in 1A. All values in the bar graphs are mean ± s.e.m. Y, young; A, aged.

Elastin haploinsufficient mice exhibit sustained blood pressure elevation, which has been attributed, at least partly, to the structural changes in conduit arteries undergoing some degree of arterial stiffening and decreased compliance (Faury et al., 2003; Le et al., 2011). Less compliant vessels typically transmit pulse waves much faster to low-impedance, high-resistive vascular beds such as in the kidney, inducing a state of chronic elevation of renal vascular resistance to maintain steady blood flow and to limit the transmission of high systemic pressure to the glomerulus (Avramovski et al., 2014; Zhang et al., 2020). Thus, increased vascular stiffening could impede renal perfusion, leading to sustained reduction in renal plasma flow and GFR. However, the causal relationship between vascular stiffening due to decreased levels of functional elastin and renal hemodynamics is unclear. Using Doppler ultrasonography, we examined how decreased levels of elastin impact impedance to blood flow to the renal vascular bed by measuring resistive index (RI) and pulsatility index (PI) at the level of the main renal artery, which reflect the overall impedance of the downstream renal vascular bed (Gosling et al., 1991). As shown by the representative waveforms of renal Doppler flow in Figure 1A, there was a pronounced increase in peak systolic and end diastolic velocities in young *Eln* ^*+/−*^ mice, and these parameters appeared to be accentuated in aged mice, regardless of genotype. Both RI and PI values were elevated in young and aged *Eln* ^+/−^ mice and trended higher in aged *Eln* ^*+/+*^ mice (Figures 1B, C). These results suggested that elastin insufficiency accelerates and enhances age-related increase in impedance to blood flow in the renal vascular bed.

### Increased impedance to RBF is associated with medial elastic fiber fragmentation

Age-related increase in vascular impedance to flow is often associated with changes in the structure of the vessel wall and is characterized by fragmentation of medial elastic fibers and vascular calcification that together contribute to increased vascular stiffness (Basalyga et al., 2004; Greenwald, 2007; Sigrist and McIntyre, 2008; Pai and Giachelli, 2010; Duca et al., 2016). Therefore, we examined whether increased impedance to RBF in *Eln* ^*+/−*^ mice, akin to age-related changes in renal hemodynamics, was associated with structural changes in the renal microvasculature. Baseline characteristics of young and aged *Eln* ^*+/+*^ and *Eln* ^*+/−*^ female mice are shown in Table 1. Body weight and kidney weight were significantly greater in aged mice relative to their young cohort. However, when normalized to tibia length or body weight, there was no difference in kidney weight among the different groups.

**TABLE 1:** Body weight and kidney weight of young and aged *Eln* ^*+/+*^ and *Eln* ^*+/−*^ mice.

Parameters	Young *Eln* ^*+/+*^	Young *Eln* ^*+/−*^	Aged *Eln* ^*+/+*^	*Aged Eln* ^*+/−*^
*n*	11	10	11	6
BW (g)	22.7 ± 0.9#	23.5 ± 0.9#	34.5 ± 1.4**	34.1 ± 4.5**
LKW (mg)	154.1 ± 8.1#	155.9 ± 9#	222.4 ± 9.7*	210.3 ± 12.7*
RKW (mg)	157.0 ± 7.7#	162.5 ± 10.0#	207.6 ± 6.9*	217.5 ± 12.9*
KW/TL ratio (mg/mm)	20.57 ± 0.8	21.3 ± 1.2	25.7 ± 0.9*	25.6 ± 2.3
KW/BW ratio (mg/g)	13.7 ± 0.5	13.6 ± 0.5	12.6 ± 0.6	13.2 ± 1.0

Values are mean ± s.e.m. *p < 0.05, **p < 0.01 within genotype comparison; ^#^ *p* < 0.05, ^##^ *p* < 0.01 across genotype comparison. BW, Body weight; LKW, left kidney weight; RKW, right kidney weight; KW/TL, kidney weight/tibia length; KW/BW, kidney weight/body weight.

**FIGURE 2:**
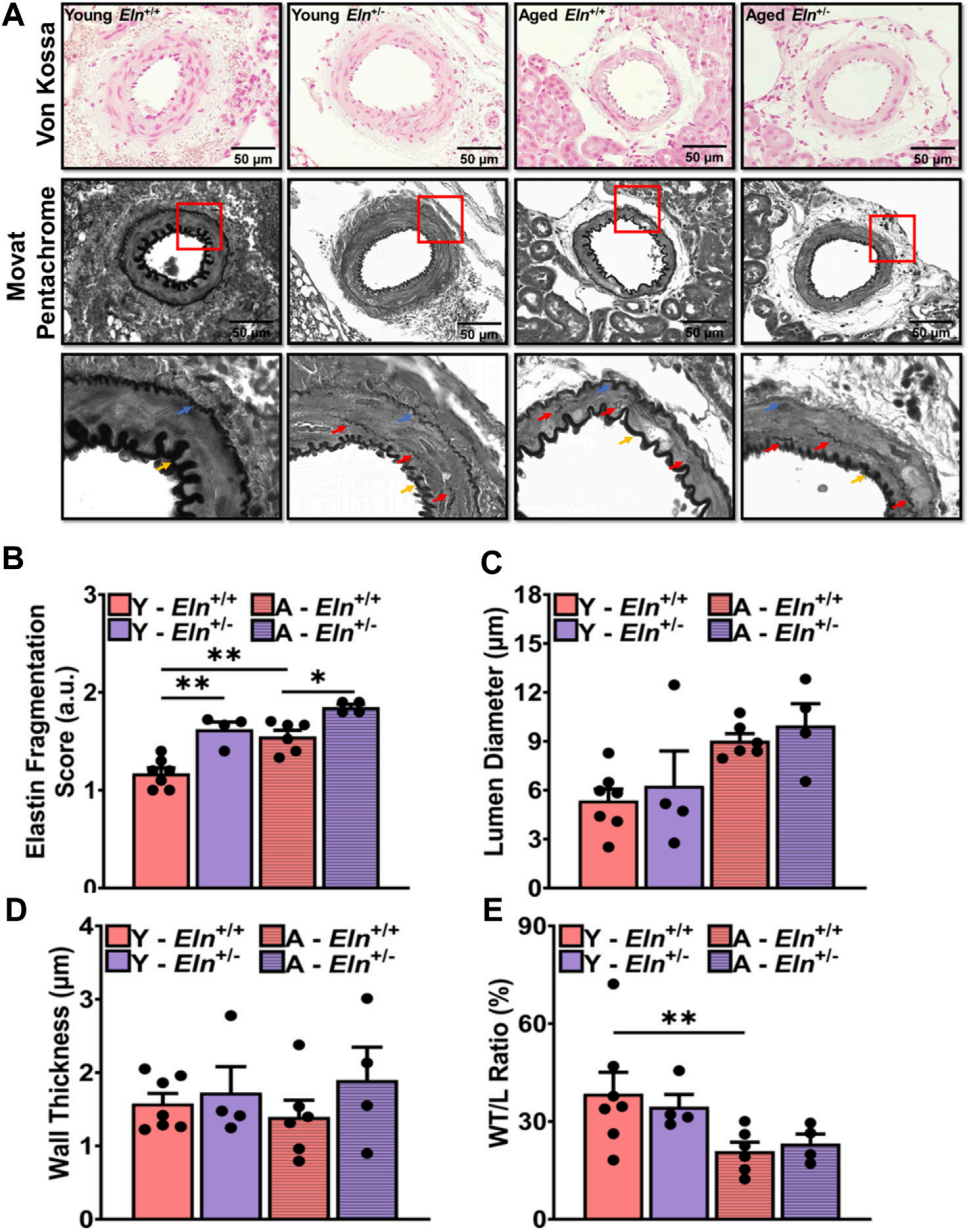
Morphological characteristics of small arteries in the renal vascular bed of young and aged *Eln* ^+/+^ and *Eln* ^+/−^ female mice. **(A)** Representative images of small intra-renal arteries with Von Kossa (top panel) and Movat Pentachrome (middle panel) staining. Movat Pentachrome images were converted to grayscale to highlight internal and external elastic laminae and fragmented elastin fibers in the medial layer. Images were acquired in brightfield at ×100 magnification using an All-in-one BZ-×800 microscope. Images in the bottom panel are the zoomed-in regions of interest highlighted by the red boxes in the middle panel. The zoomed-in images highlight the distribution of fragmented elastin in the medial layer of small intra-renal arteries as well as changes in the internal and external elastic laminae. Blue arrows point to the external elastic lamina; yellow arrows point to the internal elastic lamina; and red arrows point to fragmented elastin fibers in the medial layer. Von Kossa staining of intact vessels in the kidney exhibited no calcification in any group. Movat staining highlights increased elastin fragmentation in the medial layer and thinner elastic laminae in young *Eln* ^+/−^ and aged mice. **(B)** Scores for elastin fragmentation in the medial layer were higher in young *Eln* ^+/−^ and aged mice. From the histology images, lumen diameter **(C)**, wall thickness, **(D)** and wall-to-lumen ratio **(E)** were quantified. Analysis of the histological images was performed with FIJI software. Scoring of elastin fragmentation was performed by individuals who were blinded to the age and genotype of the mice. N = 3–7 vessels per animal from 4–6 animals per group. All values in the bar graphs are mean ± sem. *p < 0.05; ***p < 0.001. Y, young; A, aged.

Histological sections of the kidney were processed for Von Kossa staining to determine whether elastin insufficiency led to calcium deposition in the medial layer of the renal microvasculature. With this histological staining, calcified areas in the medial layer usually appear dark brown (Sakata et al., 2003). However, among the different genotypes and age groups examined in this study, there was no indication of medial layer calcium deposition (Figure 2A, top panel). We then examined the morphology and organization of elastin fibers in the medial layer of small arteries in Movat pentachrome-stained kidney sections. Because Movat pentachrome dye stains multiple structures, including elastin and collagen, in multiple colors, we converted the images to grayscale to highlight the distribution of elastin fibers, which appear as dark lines in the intimal, medial, and adventitial layers. Fragmented elastin fibers were primarily visible in young *Eln* ^+/−^ mice as well as in aged *Eln* ^+/+^ and *Eln* ^+/−^ mice (Figure 2A, middle panel, Figure 2B). Additionally, young *Eln* ^+/−^ and aged mice exhibited thinner internal and external elastic laminae. There was a trend towards increased lumen diameter in young *Eln* ^+/−^ and aged mice, while wall thickness appeared similar among the groups but showed decreasing trend in aged *Eln* ^+/+^ mice (Figures 2C, D). Wall thickness-to-lumen ratio was slightly reduced in young *Eln* ^+/−^ and aged mice of both genotypes (Figure 2E).

Overall, these data indicate that increased impedance to RBF in elastin insufficiency is associated with early structural remodeling of small arteries in the renal vascular bed, which appears to worsen with age.

### Elastin insufficiency impairs vascular recoil efficiency of renal interlobar arteries

**FIGURE 3:**
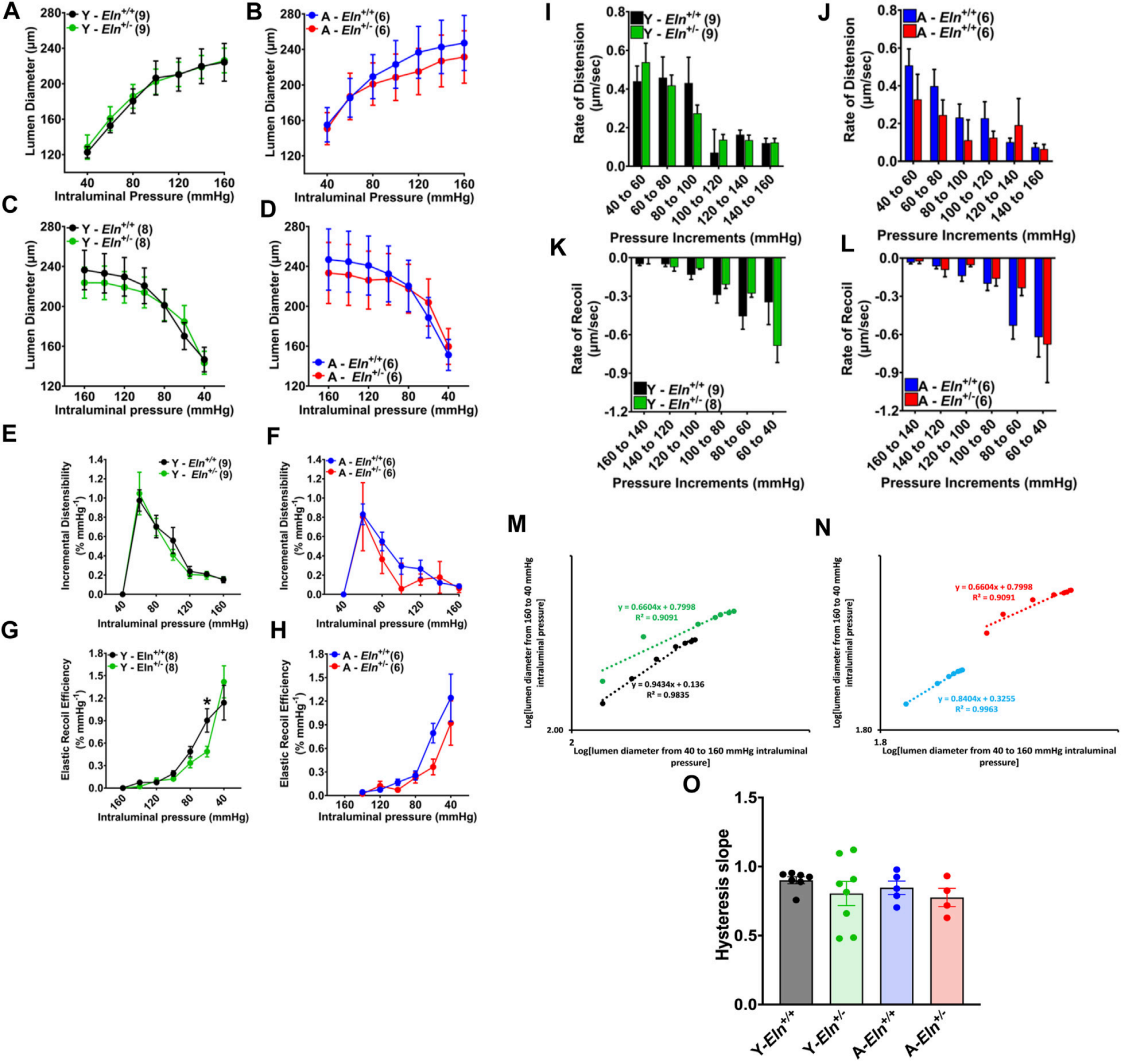
Effects of elastin insufficiency and aging on the biomechanical properties of renal interlobar arteries of young and aged *Eln* ^+/+^ and *Eln* ^+/−^ female mice. **(A**–**D)** Passive response of interlobar arteries to pressure loading and unloading in young and aged *Eln* ^+/+^ and *Eln* ^+/−^ female mice. Intraluminal pressure in 20 mmHg increments or decrements was applied to cannulated interlobar arterial segments in Ca^2+^ free HEPES buffer at 37°C. **(E**–**H)** Incremental distensibility and elastic recoil efficiency of interlobar arteries from young and aged *Eln* ^+/+^ and *Eln* ^+/−^ female mice in response to increasing or decreasing intraluminal pressure. **(I**–**L)** Rates of lumen diameter changes in response to increases and decreases in intraluminal pressure in interlobar arteries from young and aged *Eln* ^+/+^ and *Eln* ^+/−^ female mice. **(M,N)** Representative correlations of interlobar artery lumen diameters as intraluminal pressure was increased from 40 to 160 mmHg (*x*-axis) and decreased back from 160 to 40 mmHg (*y*-axis). The slope of the trendlines (dotted lines) indicates the level of hysteresis. **(O)** A summary bar graph showing the average slope of the correlation graphs illustrated in M for arteries from young and aged *Eln* ^+/+^ and *Eln* ^+/−^ mice. All values are mean ± s.e.m. *p < 0.05. The number of animals per group is 6-8 mice. Y, young; A, aged.

Impedance in a non-stenosed renal artery, as measured by Doppler ultrasound, reflects the overall impedance exerted mostly by preglomerular arterioles, including multi-layer small muscular vessels preceding afferent arterioles (Gosling et al., 1991). Conversely, renal vascular resistance is determined primarily by the tone of the renal afferent and efferent arterioles that ultimately control plasma flow to the glomerulus and glomerular pressure driving GFR (Burke et al., 2013; Carlstrom et al., 2015; Kaufman et al., 2021). Whereas vascular tone in the afferent arteriole is determined by myogenic response to changes in renal perfusion pressure and tubuloglomerular feedback mechanisms, vascular tone in the preceding small arteries and arterioles is largely determined by myogenic response moderated mostly by reactivity to vasoactive GPCR agonists, including angiotensin II and norepinephrine (Jernigan and Drummond, 2006; Hercule et al., 2007; Harhun et al., 2010; Owens et al., 2017). As shown in the preceding results, these small muscular intra-renal arteries contain elastin fibers that likely confer some level of compliance influencing the rate of cycling of these small vessels between constriction and dilatation (low hysteresis) in response to the pulsatility of renal perfusion pressure. However, the extent to which elastin insufficiency or aging impacts the biomechanical properties of these small arteries and their contribution to the overall regulation of vascular impedance to RBF in response to fluctuations in renal perfusion pressure is unknown. Therefore, first, we examined how elastin insufficiency impinges on the rate at which aging affects the passive biomechanical properties and compliance of the renal microvasculature. Using an *ex vivo* vessel preparation, isolated renal interlobar arteries from young and aged *Eln* ^+/+^ and *Eln* ^+/−^ mice were subjected to a cycle of stepwise increases (loading) and decreases (unloading) in intraluminal pressure to examine the overall arterial compliance. As shown in Figure 3A, the pressure-diameter curves were similar between young *Eln* ^+/+^ and *Eln* ^+/−^ mice during pressure loading; However, in aged mice, *Eln* ^+/−^ interlobar arteries appeared slightly less distended relative to arteries from *Eln* ^+/+^ mice at high intraluminal pressure levels (Figure 3B). During pressure unloading, interlobar arteries from young *Eln* ^+/−^ mice appeared to remain distended, as lumen diameter was somewhat unchanged with decreasing intraluminal pressure (Figure 3C). In aged mice, arteries from *Eln* ^+/−^ mice also displayed a similarly refractory response to decreasing intraluminal pressure as in young *Eln* ^+/−^ mice (Figure 3D). Second, we assessed vascular incremental distensibility as the percent change in lumen diameter per unit increase in intraluminal pressure during pressure loading, and elastic recoil efficiency as the percent change in lumen diameter per unit decrease in intraluminal pressure during pressure unloading. We found that incremental distensibility equally decreased in both young and aged mice of both genotypes (Figures 3E, F). However, during pressure unloading, arteries from young and aged *Eln* ^+/−^ mice exhibited less elastic recoil efficiency compared to their respective *Eln* ^+/+^ cohorts (Figures 3G, H). We then analyzed and compared the rate of change in lumen diameter as intraluminal pressure was increased or decreased. During pressure loading, the rate of diameter increase was similar in young animals, whereas it was somewhat decreased in arteries from aged *Eln* ^+/−^ mice (Figures 3I, J). However, arteries from both young and aged *Eln* ^+/−^ mice showed decreased rates of recoil, except when intraluminal pressure was reduced from 60 to 40 mmHg, at which point vascular recoil rate trended higher in arteries from young *Eln* ^+/−^ mice (Figures 3K, L). To assess the level of vascular hysteresis due to aging and elastin insufficiency, we plotted lumen diameter during pressure decrease from 160 to 40 mmHg *versus* lumen diameter during pressure increase from 40 to 160 mmHg. As shown in Figures 3M–O, the trendline slopes of *Eln* ^+/−^ and aged *Eln* ^+/+^ were more blunted relative to young *Eln* ^+/+^ arteries, suggesting more hysteresis in these vessels. Taken together, these data suggest that decreased functional elastin in the renal microvasculature worsens vascular hysteresis of interlobar arteries.

### Loss of functional elastin blunts renal hemodynamic response to acute increase in perfusion pressure

**FIGURE 4:**
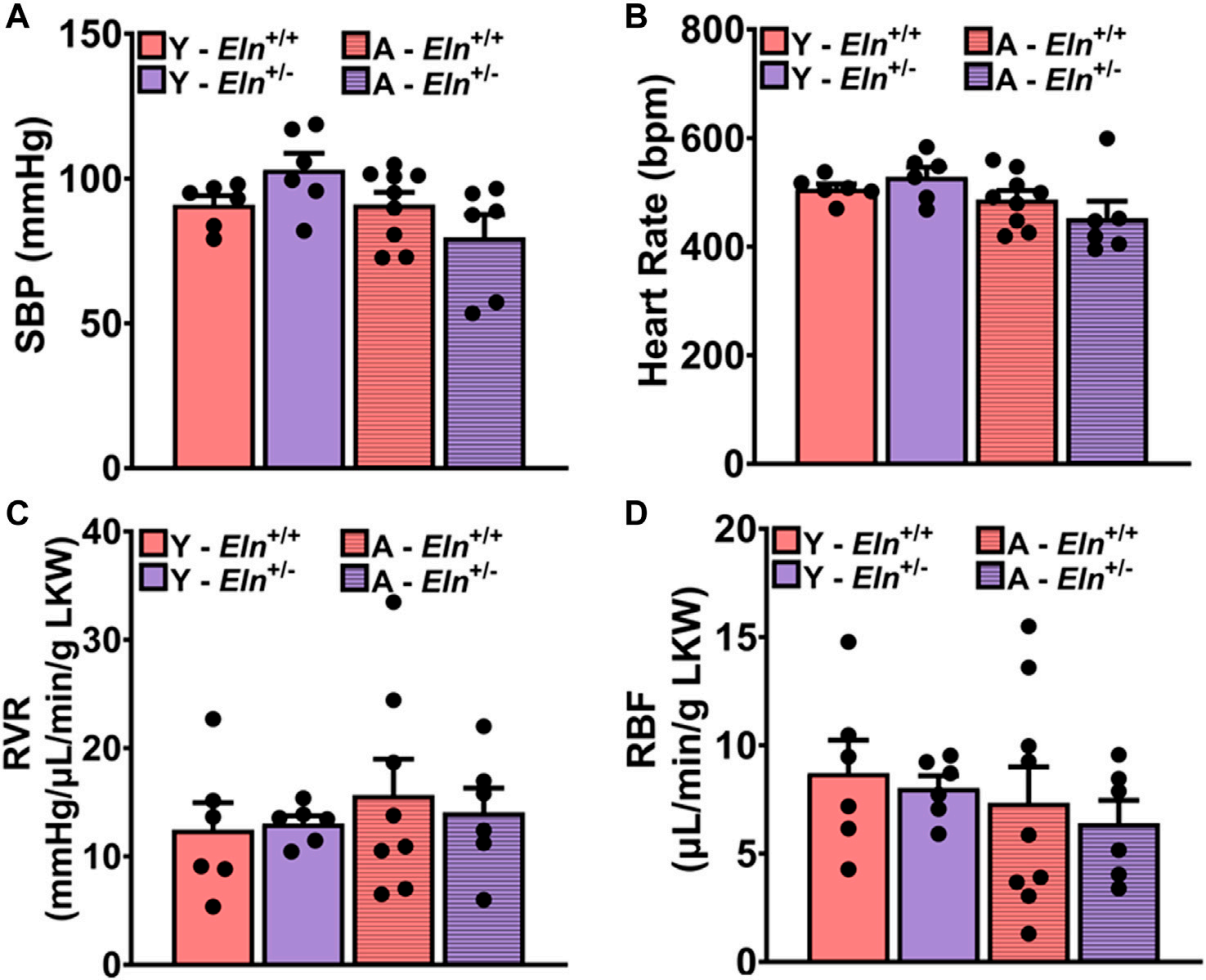
Baseline systolic blood pressure [**(A)**, SBP], heart rate [**(B)**, HR], renal vascular resistance [**(C)**, RVR], and renal blood flow [**(D)**, RBF] in young and aged *Eln* ^+/+^ and *Eln* ^+/−^ mice during systemic clamping of neurohumoral input to the kidney with a continuous infusion of a vasoactive cocktail containing 1 μg/mL norepinephrine, 0.5 ng/mL arginine vasopressin, 0.2 mg/mL hydrocortisone, and 0.2 μg/mL aldosterone in phosphate-buffered saline/albumin solution. Data are presented as mean ± s.e.m. Two-way analysis of variance with Sidak *post hoc* test was performed to compare all groups. N = 6–9 animals per group. Y, young; A, aged; LKW, left kidney weight.

Having observed the impairment of interlobar artery recoil efficiency due to elastin insufficiency, we postulated that changes in the biomechanical and structural properties of the renal vascular bed *per se* likely alter renal hemodynamics, thereby impairing renal vascular response to increased perfusion pressure to maintain steady RBF. To test this hypothesis, we examined RBF at baseline and in response to a sudden increase in renal perfusion pressure under a condition wherein neurohumoral input to the kidney was clamped by a continuous intravenous infusion of a phosphate-buffered saline (PBS) cocktail containing 2% bovine serum albumin, 1 μg/mL norepinephrine, 0.5 ng/mL arginine vasopressin, 0.2 mg/mL hydrocortisone, and 0.2 μg/mL aldosterone, as previously described (Noonan et al., 2005; Osei-Owusu et al., 2015). At baseline and prior to a sudden increase in renal perfusion pressure, systolic blood pressure (SBP) was slightly elevated in young *Eln* ^+/−^ mice, while it was slightly reduced in aged *Eln* ^+/−^ mice compared to *Eln* ^+/+^ mice of the same respective ages. At the same time, heart rate was similar between the two genotypes (Figures 4A, B). Renal vascular resistance (RVR) was slightly elevated in young *Eln* ^+/−^ mice and appeared enhanced with age relative to *Eln* ^+/+^ mice of similar ages (Figure 4C). Renal blood flow was similar between the genotypes at a young age but trended higher in aged *Eln* ^+/+^ mice but lower in *Eln* ^+/−^ mice under the condition of clamping neurohumoral input to the kidney (Figure 4D).

**FIGURE 5:**
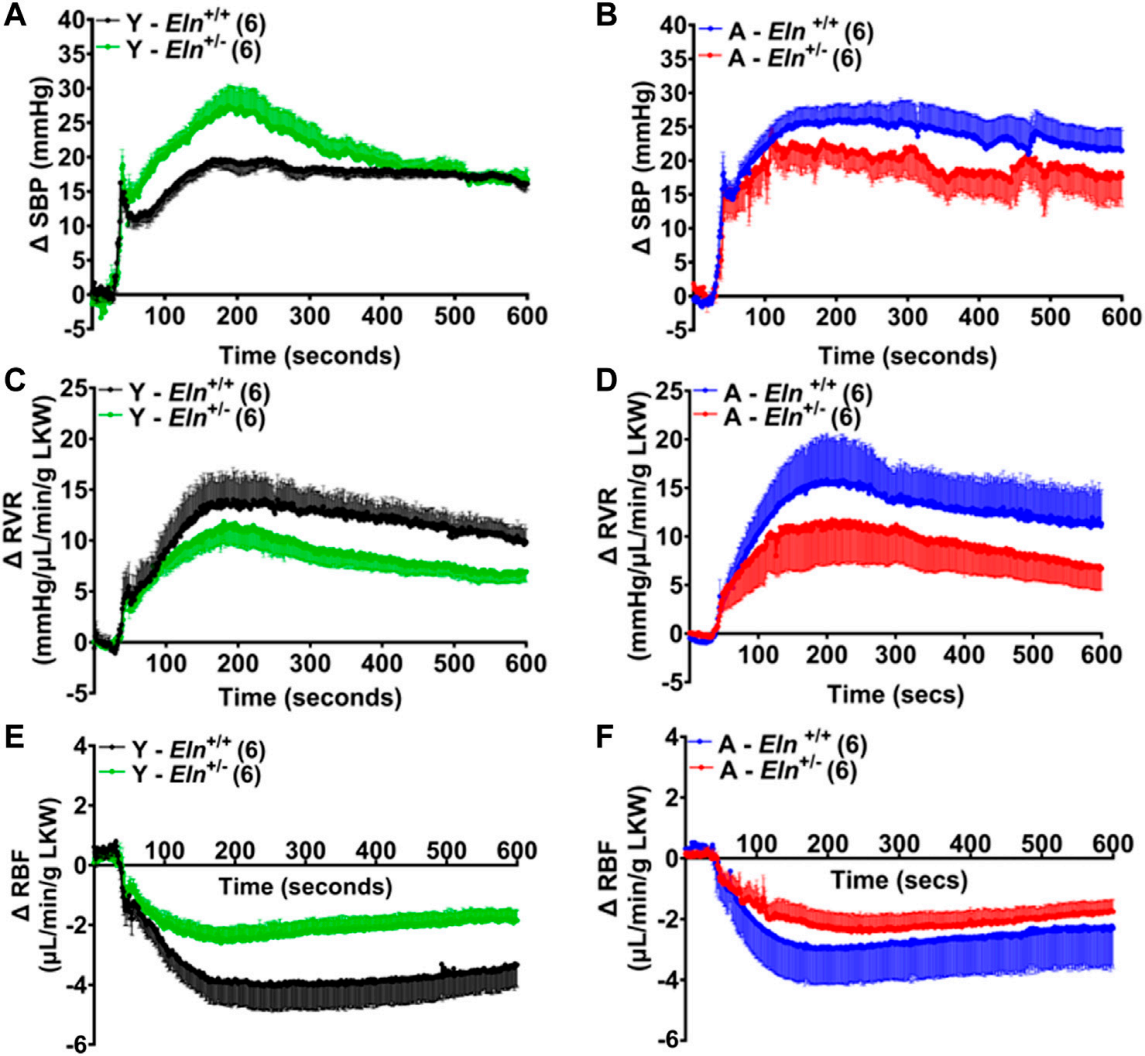
Time course of blood pressure and renal hemodynamic response to simultaneous occlusion of the celiac and superior mesenteric arteries. Blood pressure increased sharply in both young **(A)** and aged **(B)** *Eln* ^*+/+*^ and *Eln* ^*+/−*^ mice. This was accompanied by increased renal vascular resistance (RVR, **(C,D)** and a pronounced decrease in renal blood flow (RBF, **(E,F)**. Data are presented as mean ± s.e.m. Y, young; A, aged; LKW, left kidney weight.

To increase renal perfusion pressure, the celiac and superior mesenteric arteries were simultaneously occluded, causing a sharp increase in blood pressure. Compared to the response in young *Eln* ^+/+^ mice, and despite reaching a higher magnitude, the rise in blood pressure was relatively slower in young *Eln* ^+/−^ mice (Figure 5A). In addition, while blood pressure elevation after the occlusion remained steady in *Eln* ^+/+^ mice, it began to decline approximately 3 min after reaching peak pressure in young *Eln* ^+/−^ mice. In aged mice, the kinetics of blood pressure increase after occlusion was similar between the two genotypes. However, the decline from the peak values was relatively more rapid in aged *Eln* ^*+/−*^ mice (Figure 5B). As expected, the increase in renal perfusion pressure caused RVR to increase, which was less robust in both young and aged *Eln* ^+/−^ mice relative to their respective *Eln* ^+/+^ controls (Figures 5C, D). Furthermore, the increase in RVR in both young and aged *Eln* ^+/−^ mice appeared somewhat transient, as the peak responses were followed by a gradual decline over the duration of the recording. Increased blood pressure and RVR following the occlusion were accompanied by a rapid decline in RBF that was less pronounced in young *Eln* ^+/−^ mice compared to *Eln* ^+/+^ mice of the same cohort (Figure 5E). Similarly, the decrease in RBF was less pronounced in aged *Eln* ^+/−^mice compared to their aged *Eln* ^*+/+*^ counterparts, though the difference was not as remarkable as in young mice (Figure 5F).

**FIGURE 6:**
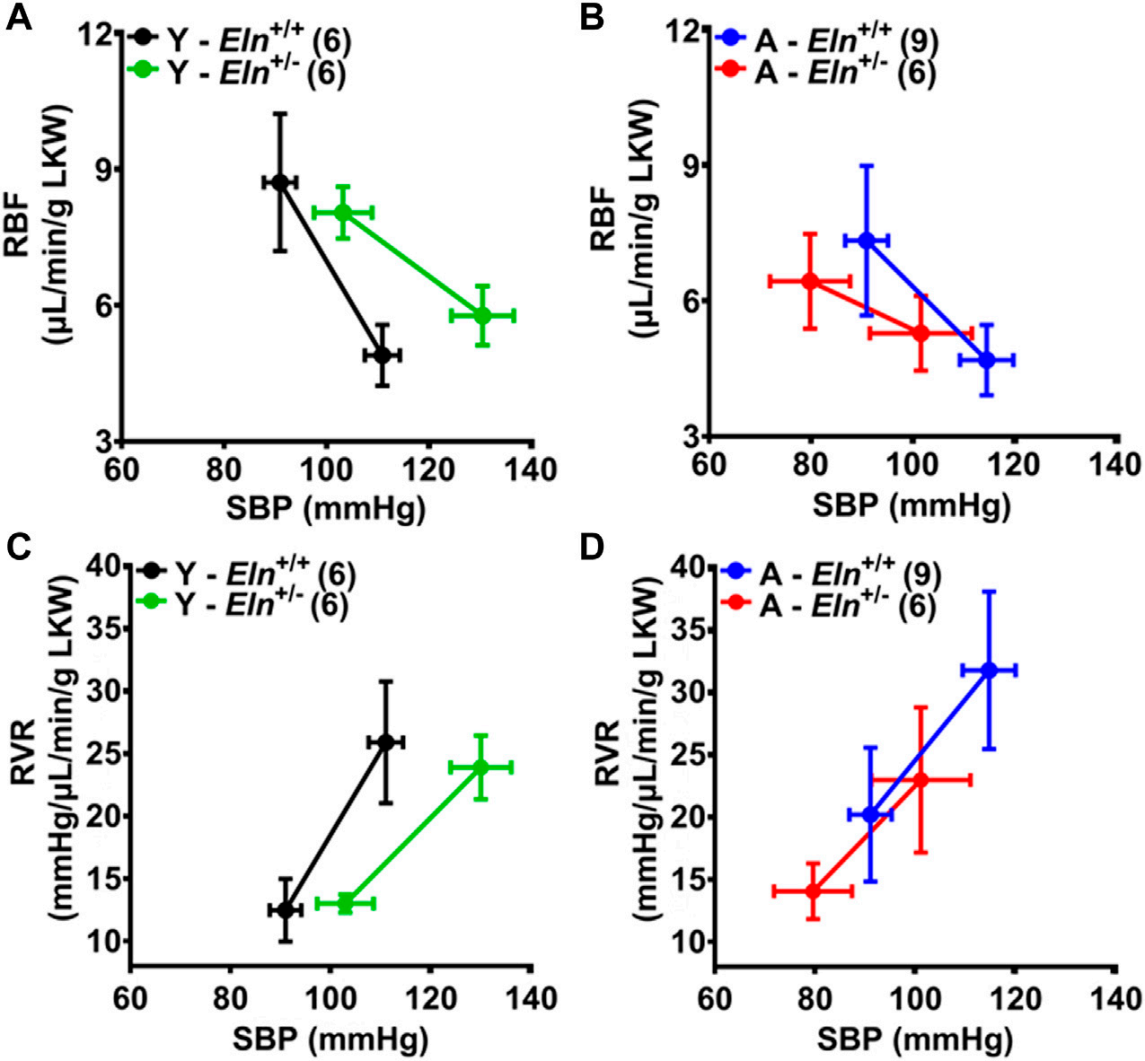
The effect of aging and elastin insufficiency on blood pressure-renal hemodynamics relationship following simultaneous occlusion of the celiac and superior mesenteric arteries. **(A,B)** Systolic blood pressure (SBP) and renal blood flow (RBF) in young and aged *Eln* ^*+/+*^ and *Eln* ^*+/−*^ mice before and after manual occlusion of the celiac and superior mesenteric arteries. **(C,D)** Renal vascular resistance (RVR) in young and aged *Eln* ^*+/+*^ and *Eln* ^*+/−*^ mice before and after a manual occlusion of the celiac and superior mesenteric arteries. Data are presented as mean ± s.e.m. Values after occlusion are the maximal SBP, RBF, and RVR responses. LKW, left kidney weight.

To further assess how elastin insufficiency and aging alter renal hemodynamics, we evaluated the MAP-RBF and MAP-RVR relationships before and after a step increase in renal perfusion pressure by plotting the maximal blood pressure, RBF, and RVR before and after simultaneously occluding the celiac and superior mesenteric arteries. Following occlusion, maximal blood pressure trended higher in young *Eln* ^+/−^ mice compared to their *Eln* ^+/+^ control; however, RBF declined to similar levels, while RBF decreased more substantially in young *Eln* ^+/+^ compared to *Eln* ^+/−^ mice (Figure 6A). In aged mice, maximal blood pressure and RBF response was similar between the two genotypes and less robust relative to the response in young mice with similar changes in RBF (Figures 6A, B). Additionally, changes in RVR and RBF in response to increased renal perfusion pressure were less robust in young and aged *Eln* ^+/−^ mice and aged *Eln* ^+/+^ mice compared to young *Eln* ^+/+^ mice (Figure 6C, D). These data indicate that vascular structural remodeling resulting from elastin sufficiency impairs the ability of the renal vasculature to respond appropriately to fluctuations in intraluminal pressure.

### Elastin insufficiency exacerbates age-related decrease in the efficiency of renal autoregulation

**FIGURE 7:**
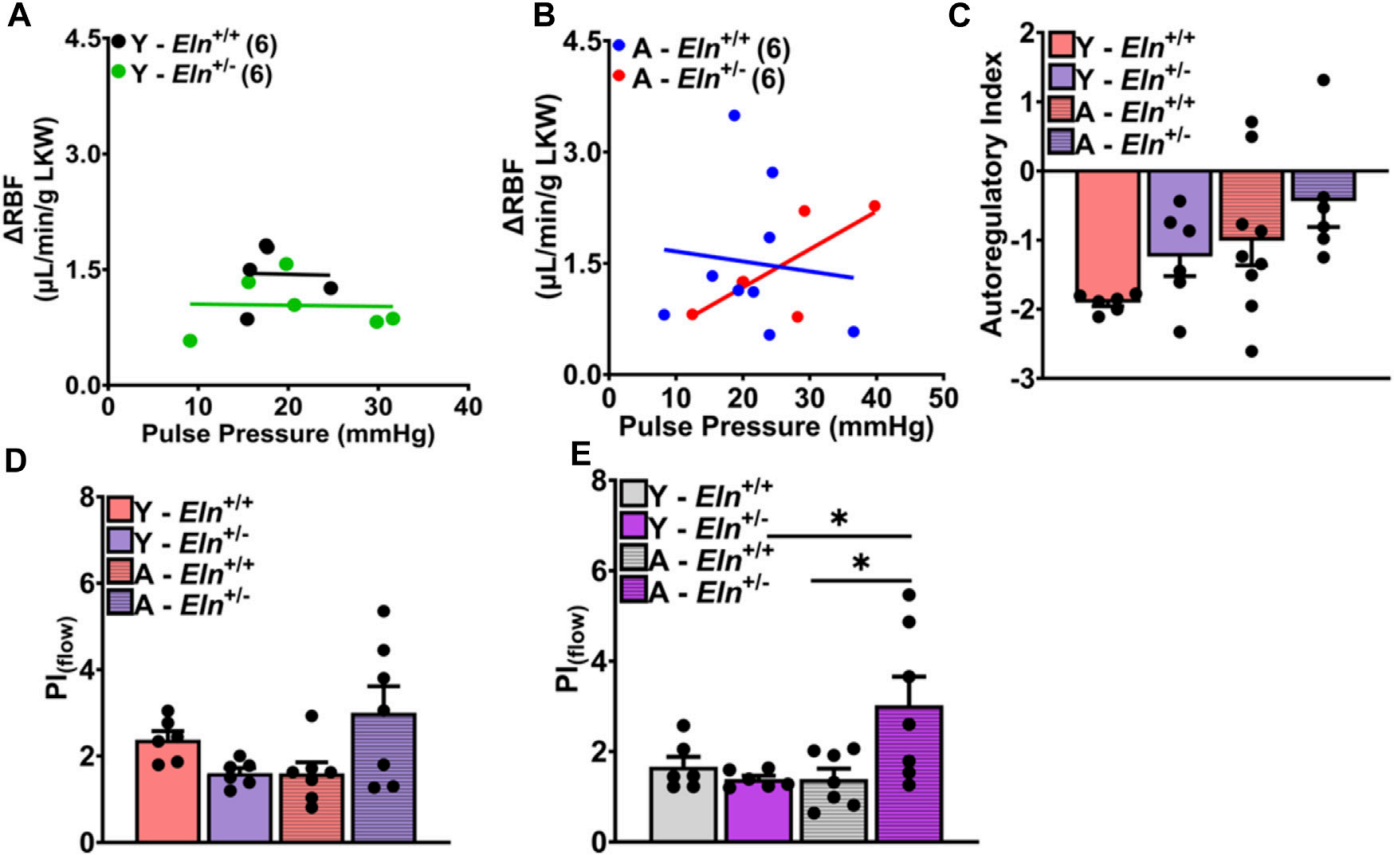
Effect of elastin insufficiency and aging on the relationship between pulse pressure and RBF and on renal autoregulatory index in young and aged *Eln* ^+/+^ and *Eln* ^+/−^ mice. Scatterplot showing the correlation between RBF and pulse pressure in young **(A)** and aged **(B)** *Eln* ^+/+^ and *Eln* ^+/−^ mice at baseline. The correlation was examined using Pearson correlation analysis with a two-tailed test; N = 5-9 animals. **(C)** Calculated autoregulation index (AI) determined using the following equation: $AI=\frac{\left[\frac{\left(RBF2-RBF1\right)}{RBF1}\right]}{\left[\frac{\left(MAP2-MAP1\right)}{MAP1}\right]},$ where RBF1 and MAP1 are the average baseline values taken after initiating continuous infusion of cocktail and 10s before a step increase in systemic pressure by manual simultaneous occlusion of the celiac and superior mesenteric arteries, and RBF2 and MAP2 are the average values at approximately 2 min after the step increase in renal perfusion pressure. **(D)** Baseline pulsatility index (PI_flow_) and **(E)** PI_flow_ after occlusion, calculated using the following equation ${PI}_{flow}=\frac{{RBF}_{\max}-{RBF}_{\min}}{{RBF}_{mean}}$ , where RBF_max_ is maximum renal blood flow, RBF_min_ is minimum renal blood flow, and RBF_mean_ is mean renal blood flow either at baseline or after occlusion. Data are presented as mean ± sem. *p < 0.05; ***p < 0.001. Y, young; A, aged; LKW, left kidney weight.

Finally, we determined whether the blunting of renal hemodynamic response to increased perfusion pressure in *Eln* ^+/−^ and aged *Eln* ^+/+^ mice with systemic clamping of neurohumoral input to the kidney was associated with changes in renal autoregulation and impedance to RBF. In aged *Eln* ^+/−^ mice, we found a strong positive correlation between pulse pressure and RBF (*r* = 0.74; *p* < 0.09), while there was a slight negative correlation between pulse pressure and RBF in aged *Eln* ^*+/+*^ mice (Figures 7A, B). Calculated average autoregulatory index (AI), a measure of the efficiency of RBF control in response to dynamic changes in systemic pressure, was closer to 0 in aged *Eln* ^*+/+*^ mice, as well as in both young and aged *Eln* ^+/−^ mice (Figure 7C). Prior to the occlusion, pulsatility index (PI_flow_), calculated using values from real-time RBF recordings, trended lower in young *Eln* ^+/−^ mice but was elevated in aged mice of the same genotype (Figure 7D). The pattern of PI_flow_ differences between *Eln* ^*+/+*^ and *Eln* ^+/−^ mice remained the same even after occlusion of the superior mesenteric and celiac arteries to elevate renal perfusion pressure (Figure 7E). Together, these results suggest that young *Eln* ^+/−^ mice display some level of impaired renal autoregulation that is worsened with age, accompanied by augmented impedance to blood flow.

## Discussion

Elastin insufficiency in aging and disease is associated with increased vascular stiffness which, by itself, is an independent predictor of cardiovascular and renal diseases (Izzo and Mitchell, 2007; Briones et al., 2010; Wagenseil and Mecham, 2012). Previous findings have shown that renal structural and functional abnormalities due to elastin haploinsufficiency precede the development of hypertension, suggesting that the loss of elastin can cause renal dysfunction, at least in animal models (Owens et al., 2017). Whereas the role of elastin in the structure and function of conduit arteries has been studied extensively (Li et al., 1998a; Li et al., 1998b; Kelleher et al., 2004; Wagenseil et al., 2005; Hirano et al., 2007; Wagenseil et al., 2010; Wagenseil and Mecham, 2012; Le et al., 2015; Lin et al., 2019), relatively little is known about the impact of the loss of this extracellular matrix protein on the structure and function of the resistance vasculature that controls organ perfusion and substantiality contributes directly to total peripheral resistance and blood pressure homeostasis (Izzo and Mitchell, 2007; Osei-Owusu et al., 2014; Owens et al., 2017). In this study, we sought to determine how elastin insufficiency affected age-related remodeling of the renal microvasculature and how those changes might contribute to age-related changes in renal hemodynamics, which is key to overall kidney function, including GFR and the maintenance of water and electrolyte balance. Our results show that elastin insufficiency accelerates aging-related increase in renal vascular impedance to blood flow associated with disorganized and fragmented elastin fibers in the medial layer of small intra-renal arteries. These structural changes in the renal vasculature accelerate the impairment of the recoil efficiency and autoregulatory responsiveness of the renal vascular bed to an acute increase in renal perfusion pressure. Together, the new findings indicate that elastin insufficiency predisposes to a faster decline in renal function associated with aging.

The renal vascular bed receives approximately 20% of total cardiac output and, under normal physiological conditions, maintains a steady blood flow by adjusting renal vascular resistance in response to changes in renal perfusion pressure (Hall et al., 2012; Hamrahian and Falkner, 2017). The afferent and efferent arterioles in the juxtaglomerular apparatus are established as the primary sites, where renal perfusion pressure and sodium chloride delivery to the macula densa trigger autoregulatory mechanisms of myogenic response and tubuloglomerular feedback to alter the diameter of these arterioles, thereby increasing or decreasing renal vascular resistance (Ren et al., 2001; Just and Arendshorst, 2003; Loutzenhiser et al., 2006; Cupples and Braam, 2007; Burke et al., 2013). Medium-sized preglomerular arteries and arterioles, including interlobular, arcuate, and interlobar arteries, proximal to the afferent arteriole are small enough to respond to fluctuations in renal perfusion pressure and pulse pressure that impact renal hemodynamics. However, the contribution of these vessels to the regulation of renal blood flow has been relatively less explored.

Like the cerebral and uteroplacental vasculature, the renal vasculature is considered a low-impedance vascular bed, with high flow but high resistivity at the afferent arterioles (Gosling et al., 1991; Feihl et al., 2009; Obeid et al., 2022). To the extent that increased vascular stiffening increases pulse wave velocity, the biomechanical properties of the vessel wall, including elasticity of medium-sized arteries and arterioles, are likely to be key determinants of the transmissibility of pulsatile flow from the aorta, via the renal artery, to the afferent arterioles for delivery of blood to the glomerulus. In cardiovascular aging, characterized by disorganized medial elastic fibers and increased stiffness of small renal arteries, the capacity of such small caliber vessels to dampen pulsatile flow decreases and become more permissive to the transmission of systemic pressure, which in turn could evoke a greater contractile response of the downstream afferent arterioles to increase renal vascular resistance and decrease renal plasma flow and GFR, or otherwise could cause barotraumatic injury to the glomerulus if these mechanisms at the afferent arteriolar level fail (Bidani and Griffin, 2004; Loutzenhiser et al., 2006; Williamson et al., 2008; Bidani et al., 2013). Previous evidence supporting this hypothesis has been obtained from non-invasive assessment of renal hemodynamics by Doppler sonography, which showed increases in the indices of impedance to flow, including resistive index and pulsatility index associated with age-related renal dysfunction (Warshauer et al., 1988; Salgado et al., 1999; Degirmenci et al., 2013). Using the same non-invasive approach in this study, we found that in young mice, elastin haploinsufficiency caused the augmentation of PI and RI as occurs in aging-induced vascular remodeling and a decline in renal function. Further examination of the renal artery waveforms from Doppler ultrasound showed that in aged mice, there is an augmented end-systolic peak and a low-end diastolic velocity, highlighting the differences in the timing of the forward-traveling wave and the reflected wave. As previously shown, a mismatch between the forward and reflected wave times suggests augmented transmission of pulsatile flow in the aorta to peripheral vessels (Georgianos et al., 2015; Georgianos et al., 2018). Consistent with this hypothesis, structural analysis of the renal microvasculature showed that young *Eln* ^*+/−*^ and aged *Eln* ^*+/+*^ mice had wider lumen diameter and thinner vessel wall, corresponding to decreased wall-to-lumen ratio. However, there was no evidence of calcium deposits as expected of vascular stiffening due to calcification (Basalyga et al., 2004; Sigrist and McIntyre, 2008; Chung et al., 2009). Instead, the outward remodeling was associated with increased fragmentation of elastin fibers in the medial layer of small arteries that was more evident in young *Eln* ^*+/−*^ and aged mice. Thus, elastin insufficiency appears to accelerate age-related changes in the structural properties of the renal vascular bed. A major functional consequence of the age-related structural changes in the small arteries and arterioles of the kidney has been suspected to be the effect on the autoregulatory capacity of the renal vascular bed (Chade, 2017). However, a compensatory increase in renal vascular resistance due to the vasoactive actions of neurohumoral factors including angiotensin II, norepinephrine, and vasopressin, could mask the ensuing pathophysiological effect of increased stiffening of the microvasculature due to abnormal changes in the wall of the microvessels.

Here, we used an experimental paradigm that enabled *in vivo* assessment of the impact of age-related changes in the biomechanical properties of the renal microvasculature on renal autoregulatory response to an acute increase in perfusion pressure. Saturation of the circulatory system with kidney-specific exogenous neurohumoral factors, by continuous infusion, ensures that cognate receptors become desensitized and unresponsive to additional surge upon changes in renal perfusion pressure or blood flow (Noonan et al., 2005). Thus, in principle, this approach minimizes the effect of extrinsic factors such that the responsiveness of the renal vasculature to increased perfusion pressure becomes dependent on vascular-intrinsic factors that mediate autoregulatory efficiency. With this experimental approach, we show that structural changes in the renal microvasculature due to aging and/or elastin insufficiency impair autoregulatory response that manifests as decreased maximal renal vascular resistance in response to increased perfusion pressure. The structural basis of the suppressed renal vascular resistance response due to elastin insufficiency is likely to be exacerbated vascular hysteresis, which would be consistent with the decreased recoil efficiency observed during pressure unloading in the *ex vivo* pressure-diameter tests using isolated renal interlobar arteries. Consistent with this postulation, a direct assessment of the correlation between vascular distention and recoil showed that elastin insufficiency and aging similarly worsened vascular hysteresis in renal interlobar arteries. Thus, the findings demonstrate that decreased compliance of the renal microvasculature due to the loss of functional elastin contributes to age-related decline in renal autoregulation that may predispose to barotraumatic injury. Furthermore, our results suggest that the need for compliance, conferred by elastic fibers, for vascular recoil during the cardiac cycle, is not limited to conduit arteries but is equally relevant in low-impedance, high-flow vascular beds, including the renal vascular bed.

In summary, we report that alterations in renal hemodynamics that manifest in aging, as seen in aged wild-type mice, are already present in young *Eln* ^*+/−*^ mice. The results demonstrate that elastin insufficiency plays a significant role in the functional and structural changes associated with aging in the resistance vasculature of the kidney. Thus, the structural and functional features of the renal vascular bed in young *Eln* ^+/−^ mice are indicative of accelerated renal vascular aging.

## Data Availability

The original contributions presented in the study are included in the article/supplementary materials, further inquiries can be directed to the corresponding author.
